# Autonomous-Strengthening Adhesive Provides Hydrolysis-Resistance and Enhanced Mechanical Properties in Wet Conditions

**DOI:** 10.3390/molecules27175505

**Published:** 2022-08-27

**Authors:** Mohammadamin Ezazi, Qiang Ye, Anil Misra, Candan Tamerler, Paulette Spencer

**Affiliations:** 1 Institute for Bioengineering Research, University of Kansas, Lawrence, KS 66045, USA; 2 Department of Mechanical Engineering, Georgia Southern University, Statesboro, GA 30460, USA; 3 Department of Civil Engineering, University of Kansas, Lawrence, KS 66045, USA; 4 Department of Mechanical Engineering, University of Kansas, Lawrence, KS 66045, USA; 5 Bioengineering Program, University of Kansas, Lawrence, KS 66045, USA

**Keywords:** dental adhesive, sol-gel reaction, self-strengthening, hydrolytic degradation, dynamic mechanical analyses

## Abstract

The low-viscosity adhesive that is used to bond composite restorative materials to the tooth is readily damaged by acids, enzymes, and oral fluids. Bacteria infiltrate the resulting gaps at the composite/tooth interface, demineralize the tooth, and further erode the adhesive. This paper presents the preparation and characterization of a low-crosslink-density hydrophilic adhesive that capitalizes on sol-gel reactions and free-radical polymerization to resist hydrolysis and provide enhanced mechanical properties in wet environments. Polymerization behavior, water sorption, and leachates were investigated. Dynamic mechanical analyses (DMA) were conducted using water-saturated adhesives to mimic load transfer in wet conditions. Data from all tests were analyzed using appropriate statistical tests (α = 0.05). The degree of conversion was comparable for experimental and control adhesives at 88.3 and 84.3%, respectively. HEMA leachate was significantly lower for the experimental (2.9 wt%) compared to control (7.2 wt%). After 3 days of aqueous aging, the storage and rubbery moduli and the glass transition temperature of the experimental adhesive (57.5MPa, 12.8MPa, and 38.7 °C, respectively) were significantly higher than control (7.4MPa, 4.3 MPa, and 25.9 °C, respectively). The results indicated that the autonomic sol-gel reaction continues in the wet environment, leading to intrinsic reinforcement of the polymer network, improved hydrolytic stability, and enhanced mechanical properties.

## 1. Introduction

The composite/tooth interface is initially sealed by a low-viscosity adhesive, but the adhesive seal is fragile and readily damaged by chemical, physical, and mechanical stresses. Damage creates crevices that are colonized by cariogenic bacteria–biodegradation by-products accumulate in the crevices and increase the virulence of cariogenic bacteria, provoking a positive feedback loop that escalates the degradation [1]. Degradation of the composite-restoration margin where the adhesive is applied can lead to secondary decay, fracture, detachment, and catastrophic restoration failure [1].

Restorative materials such as methacrylate-based dental adhesives can undergo chemical degradation in the wet, oral environment [2,3,4,5]. The degradation is prompted by the intrinsic tendency of these adhesives to absorb water that plasticizes the polymer and promotes hydrolysis of the ester bonds [6]. Degradation of the ester bonds can lead to gaps at the composite/adhesive/tooth interface [7,8]. Under in vivo conditions, it is difficult, if not impossible, to repair the adhesive [6]. With the increased use of dental composites in restorative dentistry [9], there is a dire need to develop dental adhesives that resist hydrolysis. Improving the physicochemical properties of dental adhesives has been identified as a promising approach to counteract hydrolytic degradation.

One approach involves reducing or eliminating the ester groups on the copolymer chains, for example by incorporating (meth)acrylamide-based adhesives [10]. The presence of nitrogen in the network structure of these adhesives as compared to the oxygen in acrylates mitigates the susceptibility to hydrolytic degradation [10]. Another strategy is to reduce the rate and extent of water that infiltrates the network. This can be realized by enhancing the crosslink density, increasing the degree of conversion, and/or by utilizing more hydrophobic monomers [11]. However, this approach may negatively affect adhesive infiltration of the wet, demineralized dentin [12]. In another line of research, adhesives with enzyme-inhibiting characteristics (e.g., polymerizable quaternary ammonium methacrylates [13]) were effective in preventing hydrolytic degradation [14]. However, implementing this approach may reduce the bond strength compared to other adhesives [15,16].

Our research group has developed methacrylate-based adhesives that capitalize on free-radical polymerization (FRP) and sol-gel reaction to provide adhesives with autonomic strengthening properties [6,17,18]. This category of novel dental adhesive demonstrates a persistent, intrinsic network reinforcement, leading to enhanced hydrolytic resistance (i.e., chemical durability against degradation due to the reaction with water) [19,20]. The alkoxysilyl functional groups of these adhesives can be substituted by hydroxyl groups upon hydrolysis in a wet condition. Subsequently, the hydroxyl groups undergo a polycondensation reaction to produce new Si-O-Si and Si-O-C covalent bonds. This results in the formation of additional crosslinks in the existing network structure, which enables improved mechanical properties and hydrolytic stability. 

While our group has confirmed [6,17,19] the beneficial effects of the self-strengthening approach, there are critical aspects that remain unresolved. Our previous work [18] investigated formulations with relatively higher crosslink densities and hydrophobicity, whereas the objective of the current investigation is to uncover the potential to maximize the FRP and sol-gel reactions by designing a hydrophilic formulation with a relatively lower crosslink density. It is hypothesized that this approach can provide benefits such as hydrophilicity, which can facilitate infiltration of the wet, demineralized dentin matrix; water delivery to the trialkoxysilyl groups in the adhesive, to enable a more effective sol-gel reaction and self-strengthening; and a relatively lower crosslink density, which can prevent early stiffening of the copolymer network (early stiffening may compromise chain mobility, free radical polymerization, and sol-gel reaction). Further, in contrast to the earlier work that studied the mechanical response of mostly the dry copolymers [18], in the current work, the mechanical properties are studied with copolymers that are completely saturated with water. It is anticipated that water saturation is more representative of the conditions that the copolymer will experience in the wet, oral environment. The clinical implication of the self-strengthening adhesive developed in this work is the enhanced hydrolytic resistance, which can mitigate gap formation at the composite/adhesive/tooth interface and reduce the probability of composite restoration failure.

## 2. Results

**Figure 1:**
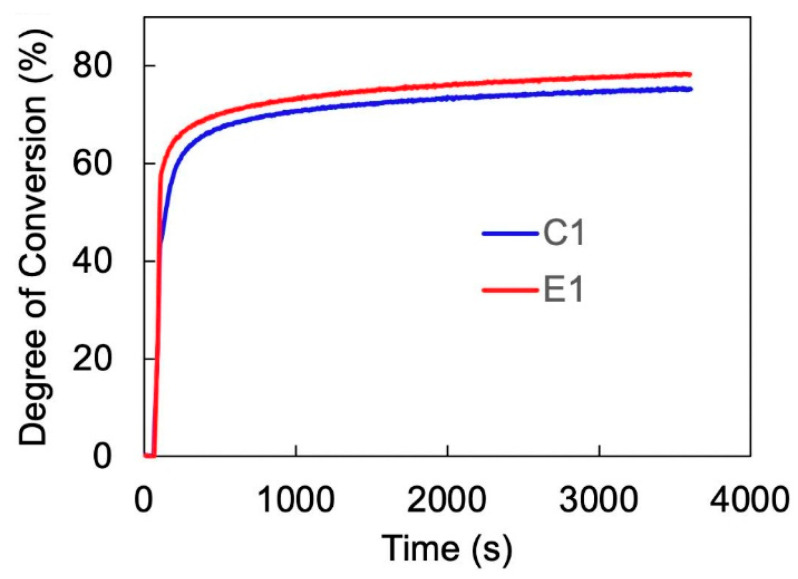
Real-time in situ degree of conversion of C=C bond.

Water miscibility tests were performed to investigate the ability of the adhesives to mix with water without showing phase separation (i.e., hydrophilicity). Results show that the water miscibility of the control and experimental formulations are approximately 26 and 25 wt%, respectively. The water miscibility of the control formulation is significantly higher than that for the experimental formulation (*p* < 0.05). Figure 1 demonstrates the real-time photopolymerization kinetic of the formulations for 1 h. The degree of conversion (DC) of the C=C double bond for the control and experimental formulations are approximately 76% and 80%, respectively. The statistical analysis demonstrates no significant difference between the calculated DC values (*p* < 0.05). The DC values further increased when the formulations were post-cured for 24 h. 

**Figure 2:**
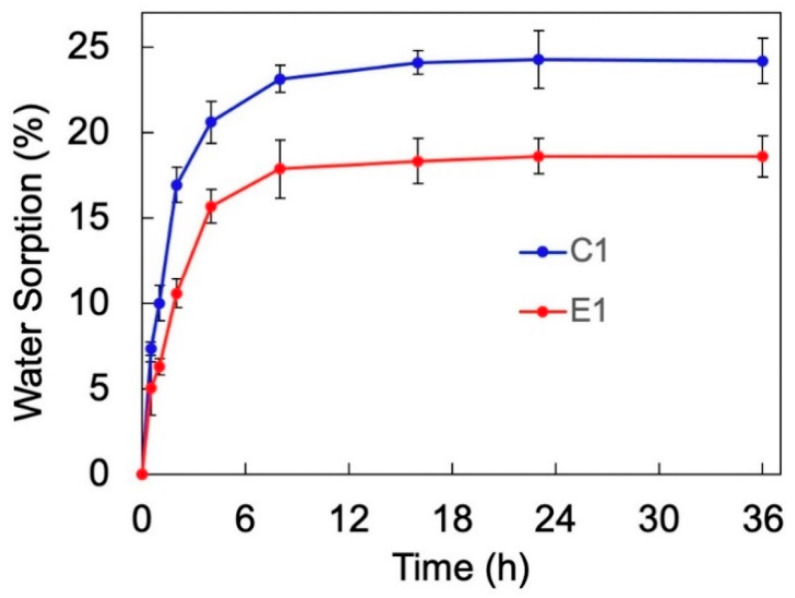
The plots of water sorption (%) as a function of storage time in water for control (C1) and experimental (E1) formulations.

Water sorption tests were also conducted to investigate the retentive capacity of the solid copolymers for water. Figure 2 demonstrates the water sorption kinetic of the control (C1) and experimental (E1) copolymers. The water sorption for both copolymers increased gradually with time and reached a plateau after approximately 24 h of storage in water. The water sorption of C1 ultimately reached approximately 24%, which is significantly higher than the ~19% demonstrated by E1 (*p* < 0.05). Table 1 lists the DC of the formulations after 1 h and 24 h of post-cure, as well as the values of *maximum polymerization rate* (*R_p_^max^*/[*M*]), water miscibility, and water sorption.

**Figure 3:**
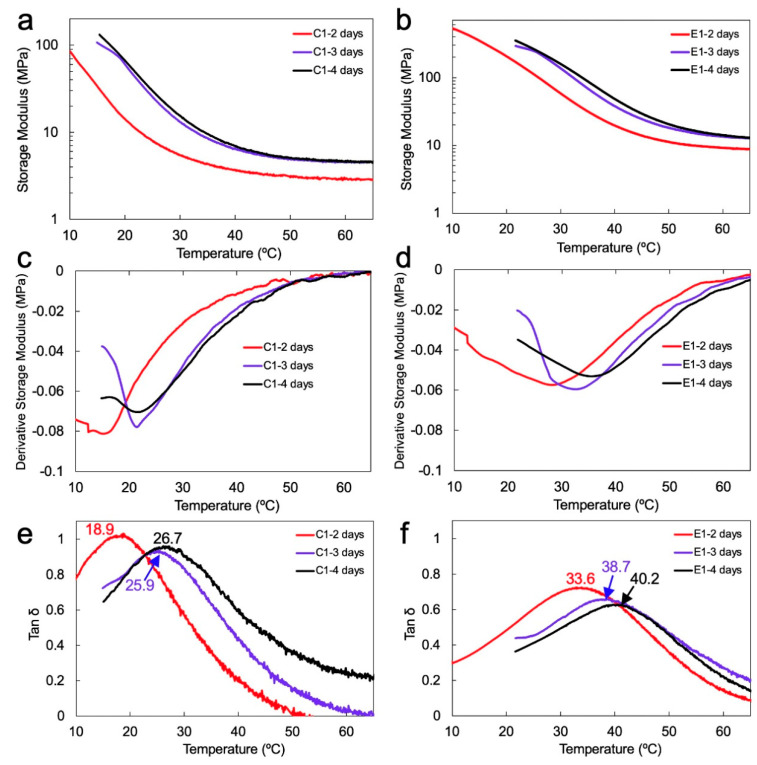
Representative plots of (**a**,**b**) storage modulus, (**c**,**d**) derivative storage modulus, and (**e**,**f**) tan δ as a function of temperature for water-saturated C1 and E1 copolymers in wet condition.

Figure 3 demonstrates the representative plots of dynamic mechanical analysis in wet condition for C1 and E1 copolymers that were aged in water (aqueous aging) for 2, 3, and 4 days. In contrast to the earlier work, which studied the mechanical response of the dry copolymers [18], this study devoted particular attention to investigating the mechanical properties of the copolymers when they are completely saturated with water. This is critical, as it mimics the load transfer in the wet oral environment [21]. In this study, the copolymers were stored in water for at least 2 days to allow sufficient time for saturation before performing the dynamic mechanical analyses (DMA) tests.

Table 2 summarizes the dynamic mechanical properties of the copolymers, including storage modulus (at 37 °C), rubbery modulus, glass transition temperature (Tg), maximum intensity of tan δ peaks, and the calculated values of relative crosslink density. The storage modulus of C1 demonstrated a significant increase from 3.9 ± 0.3 to 7.4 ± 0.4 MPa when the aqueous aging time was extended from 2 to 3 days (*p* < 0.05). 

Further aqueous aging did not result in any significant increase in the storage modulus of C1 (Figure 3a). Likewise, with the increase in the aqueous aging time from 2 to 3 days, the storage modulus of E1 increased significantly from 26.3 ± 1.0 to 57.5 ± 9.9 MPa (*p* < 0.05). Extending the aqueous aging to 4 days did not cause any significant increase in the storage modulus of E1 (Figure 3b). These results indicate that at all aqueous aging times, the storage moduli of E1 are significantly higher than those of C1.

The rubbery modulus value of C1 increased approximately 1.6 MPa when the aqueous aging time increased from 2 to 3 days (see Table 2). The E1 copolymer demonstrated an approximately 3.8 MPa increase in the rubbery modulus when the aqueous aging was extended from 2 to 3 days. The rubbery modulus of E1 after 4 days of aqueous aging remain comparable with that after 3 days (*p* < 0.05). Similar to the storage modulus behavior, the rubbery moduli values of E1 at all aqueous aging times are significantly higher than those demonstrated by C1 (*p* < 0.05).

**Figure 4:**
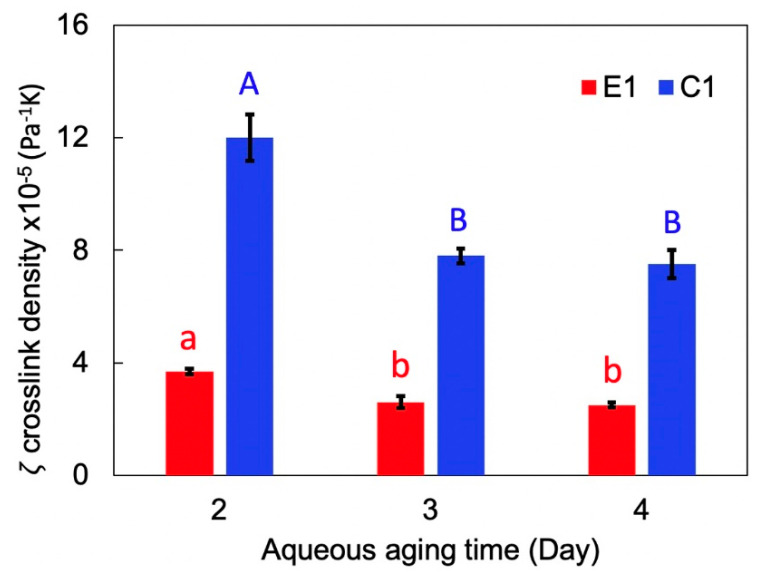
Relative crosslink density of water-saturated copolymers in wet condition. Same letter indicates no statistically significant difference (*p* < 0.05).

From tan δ plots (Figure 3e,f), the Tg values for C1 increased significantly from 18.9 ± 0.4 to 25.9 ± 0.9 °C after 3 days of aqueous aging and remained nearly constant. For E1, the values of Tg increased significantly from 33.6 ± 0.6 to 38.7 ± 0.8 °C after aqueous aging for 3 days. Extending the aqueous aging to 4 days resulted in a further increase in Tg to 40.2 ± 0.1 °C. Statistical analysis revealed that at all aqueous aging times, the Tg values exhibited by E1 are significantly higher than those for C1. The resulting increase in the copolymer network density with the aqueous aging time, as revealed mainly by E1, is assumed to lower the mobility of side chains [6]. This is supported by the maximum intensity of tan δ peaks that decreased with the increase in the aqueous aging (Figure 3e,f and Table 2). Figure 4 shows the calculated values of relative crosslink density (ζ). Results indicate a significant decrease in the values of ζ for both C1 and E1 when extending the aqueous aging time from 2 to 3 days. Note that a decrease in ζ values indicates an increase in the crosslink density.

**Figure 5:**
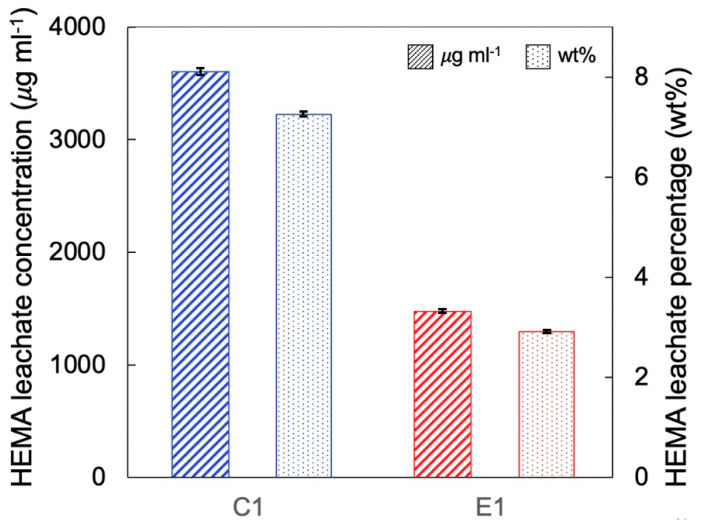
Cumulative concentration and weight percentage of HEMA leachate that was released from C1 and E1 samples after 4 days of aqueous aging.

Figure 5 demonstrates the plots of cumulative concentration and weight percentage of 2-hydroxyethyl methacrylate (HEMA) leachate released from the copolymers while undergoing aqueous aging in water at human body temperature (37 °C) for 4 days. By comparing the intensity of the HEMA peaks in the chromatograms of the storage solutions with the calibration curves of standard HEMA solutions, the cumulative HEMA leachate concentrations were calculated as 3606 ± 25 and 1473 ± 12 μg mL^−1^ for C1 and E1, respectively. The mean values are significantly different at the 0.05 level. The weight percentage of the HEMA leachate with respect to the total HEMA was calculated as 7.2 ± 0.05 and 2.9 ± 0.02 wt% for C1 and E1, respectively. Note that HEMA was the main component appearing on the chromatograms, whereas other monomers (bisphenol A glycerolate dimethacrylate (BisGMA), methacryloxyethoxytrimethylsilane (MES), and γ-methacryloxypropyl trimethoxysilane (MPS)) were not detected.

## 3. Discussion

Phase separation of methacrylate-based dental adhesive is a major limitation that can compromise the integrity and durability of the adhesive bond layer. When the adhesive infiltrates the wet, demineralized dentin structure (the structure is primarily type I collagen, water, and isolated mineral), it can phase separate into hydrophobic- and hydrophilic-rich phases [12,22]. The major components within the hydrophobic-rich phase are the crosslinker (BisGMA) and the photoinitiators (camphoroquinone (CQ), ethyl-4-(dimethylamino) benzoate (EDMAB), and diphenyliodonium hexafluorophosphate (DPIHP)), whereas HEMA and water are the main constituents of the hydrophilic-rich phase [23,24]. This indicates a compromised degree of conversion within the hydrophilic-rich phase. Consequently, the hydrophilic-rich or low-crosslinked phases facilitate diffusion of water, resulting in degradation of the bonds at the adhesive interface with the dentin [25].

In addition to phase separation, following photopolymerization, the composite/adhesive/tooth interface can be compromised as a result of chemical hydrolysis [5,26]. Bacterial growth can be promoted in the presence of the degradant byproducts [27,28,29]. It is evident that adhesive bond failure at the composite/tooth interface can trigger a cascade of events that ultimately result in recurrent decay and failure of the composite restoration [20].

To address the hydrolytic instability of dental adhesives, several strategies have been proposed that involve alterations to physicochemical properties of the adhesives, for example by tailoring the hydrophobicity [11,30,31,32], enhancing the monomer conversion at the hybrid layer [33,34,35,36], or addition of inhibitors [14,37,38]. Despite the promise of these methodologies, the intrinsic tendency of the methacrylate-based dental adhesives to water sorption renders the hydrolytic degradation inevitable in a wet oral environment [9]. Alternatively, the dental adhesives that can demonstrate the self-strengthening property have enabled improved hydrolytic stability [6,17,18,19]. However, prior work has devoted limited attention to systematically investigating the properties of self-strengthening adhesives under mechanical and physical loading when the adhesives are completely saturated with water. This is critical because the sol-gel reaction that enables the self-strengthening proceeds at a relatively slow rate [9,17]. Further, testing the durability of these adhesives in a wet condition mimics their physical and mechanical response in the oral environment.

### 3.1. Hydrophilic Adhesive Formulations, Water Miscibility, and Sorption

The adhesive formulations utilized in this study comprise 73 wt% HEMA, which has an abundance of hydrophilic hydroxyl (-OH) group. In addition, with only 15 wt% BisGMA, the proposed formulations were assumed to demonstrate a relatively low crosslink density. Such relatively low-crosslink hydrophilic formulations were expected to provide the following benefits: (1) hydrophilicity can facilitate delivery of water to the trialkoxysilyl functional groups of the MPS in E1 formulation; (2) delivery of water to these groups will facilitate a more effective and accelerated sol-gel reaction and self-strengthening; (3) a relatively low crosslink density can potentially prevent early copolymer network stiffening, which can interfere with chain mobility, free-radical polymerization, and sol-gel reaction. 

While the hydrophilicity of the formulations can facilitate infiltration into the wet dentin structure, it can accelerate the sorption of water in a wet environment after polymerization. In an ideal condition, the adhesive network should remain insoluble with water while demonstrating a relatively high hydrolytic stability [39]. The results from water miscibility tests show that the control formulation has a potential to infiltrate the wet dentin structure more effectively, as it demonstrates higher water miscibility (25.8 ± 0.4 wt%) compared to the experimental formulation (24.6 ± 0.2 wt%). Note that the adhesive formulations demonstrate turbidity above their water miscibility threshold due to phase separation as described by water-adhesive ternary phase diagram [25,40]. From water sorption results, an accelerated sorption behavior can be observed during the initial 8 h of testing (see Figure 2). This can be attributed to the water diffusion [41] to the copolymer network, which is accelerated due to the large initial water gradient. Further, the water sorption results indicate that E1 has a significantly lower retentive capacity for water than C1. This is attributed to the stiffening characteristic of E1 that proceeds with the evolution of new crosslinks within the network structure as a result of sol-gel reaction [18].

### 3.2. Polymerization Behavior

The degree of conversion is an important factor that can affect the quality of the bond at the adhesive/dentin interface [19]. The free radical polymerization of methacrylates has been extensively investigated in the prior literature [42,43,44]. When the adhesive is irradiated by visible light, the amine free radicals are generated via the transfer of electron-protons between the excited CQ and EDMAB. Consequently, the generated free radicals enable the polymerization of methacrylate monomers [17]. In the current study, the conversion of the methacrylic double bond was monitored by using the band ratio profile 1637 cm^−1^ (C=C)/1715 cm^−1^ (C=O) [45]. Our results show that the real-time DC values of C1 and E1 are comparable at approximately 76 and 80%, respectively (see Figure 1). This indicates that the difference in the type of organosilanes (MES and MPS) had a negligible effect on the conversion of C=C double bonds. When the copolymers post-cured for an extended period (24 h) in a dark ambient condition, the DC reached even higher values (see Table 1). This can be attributed to the FRP reaction, which can continue even after the visible light irradiation. Note that the conversion of C=C double bonds in methacrylates rarely reaches 100% because the crosslinked network restricts the propagation and mobility of free radicals [19]. Consequently, the incomplete conversion of double bonds can give rise to the release of leachates, such as HEMA, which increases the risk of cytotoxicity. Hence, introducing additional crosslinking by sol–gel reaction can ultimately contribute to a lower risk of cytotoxicity.

### 3.3. Role of Water in Promoting Sol-Gel Reaction and Self-Strengthening

Dynamic mechanical analysis using a three-point bending submersion clamp was implemented to capture the effect of sol-gel-driven self-strengthening on the mechanical properties of water-saturated copolymers. Considering the DMA tests were performed in submerged condition, the results were expected to better represent the mechanical response in a wet oral environment [17]. Furthermore, a previous study [18] devoted limited attention to performing DMA tests on the copolymers that are completely saturated with water. This is critical because the simultaneous water diffusion to the copolymer and mechanical loading can result in abnormally high creep strains or lower strength levels [17]. In the present study, the DMA tests were performed on the copolymers that had been submerged in water for at least 2 days. This ensured that the copolymers absorb sufficient water to reach complete saturation level. Note that the water sorption results demonstrated a plateau after approximately 24 h for both C1 and E1, indicating a state of equilibrium and saturation (see Figure 2). 

The storage moduli of C1 and E1 are demonstrated in Figure 3a,b and Table 2. While the DC values of C1 and E1 are comparable (see Table 1), the storage moduli of E1 are significantly higher than those demonstrated by C1 irrespective of the aqueous aging time. These results are attributed to the sol-gel reaction that is promoted in the wet environment and enables the formation of new crosslinks in the E1 copolymer’s network. The result of the newly formed crosslinks is the self-strengthening that enables improved mechanical properties. Such sol–gel-driven self-strengthening occurs as a result of hydrolyzing the alkoxysilyl groups to silanols in a wet environment, followed by polycondensation reaction that forms Si-O-Si and Si-O-C covalent bonds [6,17]. Note that the storage moduli increased significantly for both copolymers upon extending the aqueous aging from 2 to 3 days. For E1, this is attributed to self-strengthening behavior as a result of sol-gel reactions [6,17]. In the case of C1, it is likely that the unreacted HEMA acted as plasticizer to contribute to a higher copolymer chain mobility. By extending the aqueous aging time to 3 days, the majority of unreacted HEMA was released to enable a stiffer copolymer network. At the same time, the increased mobility of the copolymer network could mobilize the trapped free radicals to polymerize unreacted C=C double bonds and enhance the crosslink density [17]. These results are supported by the rubbery moduli values, which increased significantly at 3 days of aqueous aging, suggesting the formation of new crosslinks (see Table 2).

Meanwhile, from derivative storage modulus plots (Figure 3c,d), a major transition peak was observed, which is related to the movement of the segments in the main copolymer chains [46]. At a given aqueous aging time, the transition peak was observed at a higher temperature for E1 as compared to C1, indirectly indicating lower mobility of E1 copolymer chains.

As demonstrated in Figure 3e,f, the values of Tg shifted significantly from 18.9 to 25.9 °C and 33.6 to 38.7 °C for C1 and E1 copolymers, respectively, when the aqueous aging was increased to 3 days. Such a significant increase in Tg agrees with the rubbery modulus behavior, which supports the reduced plasticizing effect of water and the increase in the network density. While the values of Tg after 3 and 4 days of aqueous aging are comparable for C1, an increase in the Tg for E1 after 4 days indicates further self-strengthening of the network by the sol-gel reaction. The self-strengthening behavior can also be inferred from the maximum intensity of tan δ peaks, which decreased with aqueous aging time, indicating lower network mobility [6] (Figure 3e,f).

To quantify the network density of copolymers, we calculated the relative crosslink density. The inverse ratio (ζ) of modulus in the rubbery region to the temperature (65 °C) was utilized in order to calculate the values of relative crosslink density for C1 and E1 copolymers [47,48]. Note that we utilized 65 °C because the modulus reached a plateau approximately at this temperature (see Figure 3a,b). Figure 4 demonstrates the calculated values of relative crosslinking density for the copolymers that were stored in water for 2, 3, and 4 days. Both C1 and E1 copolymers demonstrated similar trends for crosslink density with the time of aqueous aging. Significantly lower values of ζ were demonstrated by both copolymers after 3 days of aqueous aging as compared to 2 days. The decreased values of ζ indicate higher crosslink density. The E1 copolymers demonstrated a more significant increase in the crosslink density compared to C1 when the aqueous aging time increased from 2 to 3 days due to sol–gel-driven formation of new crosslinks. Note that a plateau in the ζ values after 3 days of aqueous aging likely suggests the completion of sol-gel reaction within 3–4 days of storage in water. Such a relatively fast sol-gel process could be the result of the combinatorial effect of the hydrophilic and relatively low-crosslink-density formulation, which prevented early copolymer network stiffening and enabled accelerated delivery of water for hydrolysis/polycondensation reactions. Note that a highly crosslinked network of a polymethacrylate-based copolymer was identified as a factor to retard the condensation of silanol groups during sol-gel process [17].

### 3.4. High-Performance Liquid Chromatography (HPLC) Studies and Mechanism of Network Structure Evolution

Water was utilized as the storage medium in this study. Although the usage of other solvents, such as ethanol, can potentially accelerate the leaching process and enable the release of hydrophobic degradants (e.g., BisGMA), ethanol is not considered a clinically relevant solvent [20]. However, future studies should explore the effect of aggressive solvents such as ethanol:water mixtures (e.g., 75:25 w:w) on the leaching process. The results from HPLC show that the cumulative HEMA leachate concentration for C1 after 4 days of aqueous aging is more than twice that demonstrated by E1 (see Figure 5). This is attributed to the sol-gel reaction in water, which enables formation of additional crosslinks between the silanol/silanol groups of MPS and silanol/hydroxyl groups of MPS/HEMA. Consequently, a lower number of HEMA molecules were released from E1 while submerged in water.

**Scheme 1:**
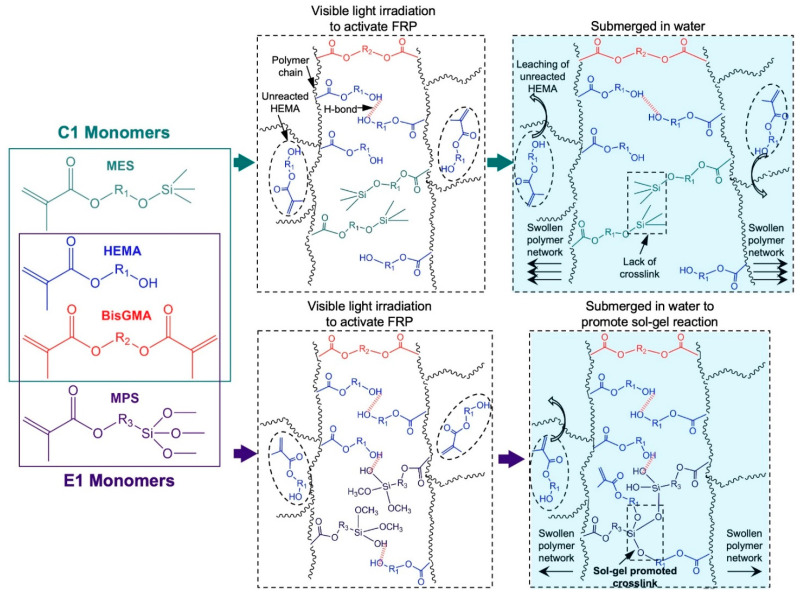
Schematic of the proposed hydrophilic polymethacrylate-based adhesives with a relatively low crosslink density. Schematic illustrates the cascade of events resuming in the C1 and E1 copolymers that includes formation of copolymer network by free-radical polymerization and release of HEMA leachate when submerged in water. Note that the sol-gel-driven self-strengthening only resumes in water for E1 copolymer due to hydrolysis-polycondensation reaction at the trialkoxysilyl functional groups of MPS.

The results of the characterization tests reveal that a cascade of events including free radical polymerization, sol-gel reaction, and leaching of unreacted HEMA contributed to the final network structure of the copolymers. These events are demonstrated in Scheme 1. The evolution of the network structure by free-radical polymerization is quite similar for both copolymers. The network structures of both C1 and E1 are primarily crosslinked by the covalent bonds through BisGMA. Additionally, the hydrogen bonds between the hydroxyl groups provided by polyHEMA side chains also contributed to the network structure of the copolymers [49]. In the case of E1, additional hydrogen bonds were also likely to be provided by MPS via partial hydrolysis of the trialkoxysilyl functional groups to the intermediate species such as -Si(OCH_3_)_2_(OH). Formation of these intermediates has been attributed to catalysis by the photoacids that are generated as a result of onium salt (DPIHP) photolysis [17]. Despite relatively weaker interaction provided by hydrogen bonds, they play important roles in tuning the chain mobility of the copolymer [18].

The evolution of network structure in the copolymers differ significantly when they are submerged in water (aqueous aging). As for C1, the trimethylsilane group in the MES lacks the ability to undergo the hydrolysis-polycondensation process and generate hydroxyl groups. This indicates that MES could not contribute to the crosslinking and network evolution via hydrogen bonding. Consequently, the relatively lower crosslink density of C1 resulted in a higher water sorption, swelling, and the release of higher concentrations of unreacted HEMA. For E1, aqueous aging resulted in the promotion of sol-gel reaction by MPS. The reaction included the hydrolysis of trialkoxysilyl groups to silanol, followed by condensation to form covalent bonds with other silanols or hydroxyl groups. The result of this chain reaction was the formation of additional crosslinks that suppressed water sorption and swelling, and lowered the release of HEMA leachate.

## 4. Materials and Methods

### 4.1. Materials

**Scheme 2:**
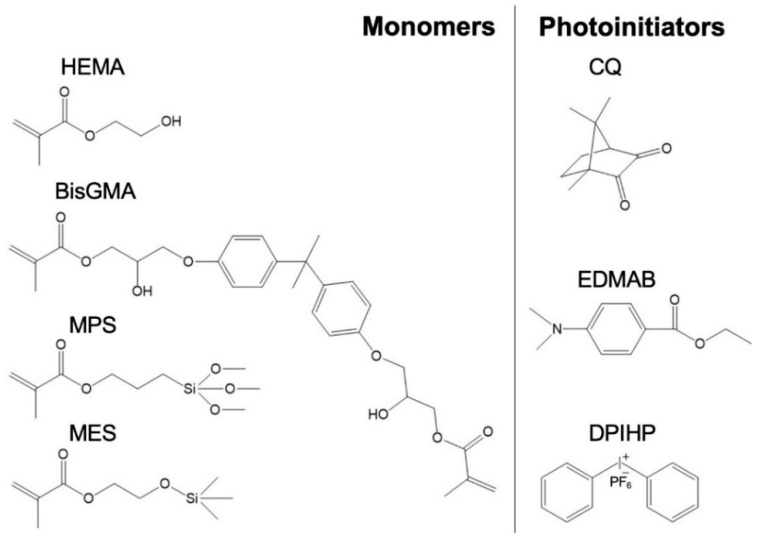
Chemical structures of monomers and photoinitiators utilized in the control and experimental formulations.

The following monomers and photoinitiators were purchased from Sigma-Aldrich (St. Louis, MO, USA): 2-hydroxyethyl methacrylate (HEMA), bisphenol A glycerolate dimethacrylate (BisGMA), diphenyliodonium hexafluorophosphate (DPIHP), camphoroquinone (CQ), and ethyl-4-(dimethylamino) benzoate (EDMAB). The organosilanes, methacryloxyethoxytrimethylsilane (MES), and γ-methacryloxypropyl trimethoxysilane (MPS) were purchased from Gelest Inc. (Morrisville, PA, USA) and MP Biomedicals (Solon, OH, USA), respectively. All materials were utilized as received without performing further purification. Scheme 2 lists the chemical structures of the monomers and photoinitiators.

### 4.2. Preparing the Adhesive Formulations

A three-component photoinitiator system consisting of DPIHP (1.0 wt%), EDMAB (0.5 wt%), and CQ (0.5 wt%) [47] were added to an amber vial. HEMA (73.0 wt%) was then added to the vial and stirred to promote complete dissolution. Subsequently, 10 wt% of the organosilanes, MES or MPS, and BisGMA (15 wt%) were added and shaken for 24 h at room temperature (23 ± 2 °C) to obtain a homogeneous adhesive solution (see Table 3).

### 4.3. Water Miscibility of Adhesive Formulations 

Approximately 500 mg of the neat adhesive was added to an amber vial. Distilled water was subsequently added to the vial in increments of 5 mg until the translucent mixture became turbid. The water percentage in the turbid mixture (*p*1) was recorded. The back titration of the turbid mixture was then performed by adding the neat adhesive until the mixture became translucent again. The water percentage in the translucent mixture (*p*2) was recorded. Three values of *p*1 and *p*2 were measured for each formulation. The average value of *p*1 and *p*2 was noted as the water miscibility. 

### 4.4. Real-Time Methacrylic C=C Bond Conversion

Fourier transform infrared spectroscopy (FTIR) was utilized for determining the degree of conversion [41]. The infrared spectrometer (Frontier FTIR Spectrometer, Perkin-Elmer, Waltham, MA, United States) was employed at 4 cm^−1^ of resolution and with a wavenumber range of 550–4000 cm^−1^ to continuously monitor the photopolymerization behavior of the adhesives in situ. The infrared spectrometer is equipped with software (Spectrum TimeBase v3.0, Perkin-Elmer, Waltham, MA, USA) that allows spectrum scans to be taken continuously. Therefore, the DC as a function of time can be determined. The conversion of methacrylic C=C double bond was monitored by using 1637 cm^−1^ (C=C)/1715 cm^−1^ (C=O) as the band ratio profile [45]. The DC values were ultimately calculated according to the equation DC = (1 − *R_p_/R_R_*) × 100, where *R_p_* and *R_R_* are the C=C/C=O band ratios for adhesive after polymerization and before polymerization, respectively. The reported value of DC is the average of the last 30 values of the time-based spectra.

Approximately 5 μL of the adhesive solution was poured on the crystal of the attenuated total reflectance (ATR) accessory (Universal ATR Sampling Accessory, Perkin-Elmer, Waltham, MA, USA). The adhesive was covered by Mylar film against exposure to ambient oxygen and moisture. The adhesive was exposed to a visible curing light (Spectrum 800, Dentsply, Milford, DE, USA) with 550 mW cm^−2^ of intensity. Note that the samples were exposed to visible light for 40 s, and the IR spectra were recorded continuously for ~3500 s after visible light activation. Three measurements were recorded for each formulation.

The maximum slope in the linear region of the DC vs. time plots was utilized to determine the maximum polymerization rate (*R_p_^max^*/[*M*]) [47].

### 4.5. Preparing Copolymer Samples 

The glass-tubing molds (Square Boro Tubing, Cat # 8100-300, VitroTubes, Mountain Lakes, NJ, USA) were vapor deposited with perfluorodecyltrichlorosilane (Thermo Scientific^TM^, Cat # L16606.18, Waltham, MA, USA) at 100 °C for 4 h in a sealed beaker. The treatment with perfluorodecyltrichlorosilane lowers the adhesion of the glass tubing’s internal wall with the adhesive and facilitates removal of the beam samples after polymerization. Subsequently, the beam samples for dynamic mechanical analysis were prepared by injecting the adhesive solution in glass tubing molds followed by curing with visible light for 40 s at 23 ± 2 °C. The rectangular beam samples (1 mm × 1 mm, length = 15 mm) were removed from molds after being stored in the dark for 1 h.

### 4.6. Water Sorption of Copolymers 

Four rectangular beam samples were prepared for each formulation, and post-curing in dark was performed for 1 h at 23 ± 2 °C. The beam samples were then stored in distilled water at 23 ± 2 °C to prewash for 4 days. Next, the samples were stored in an incubator (Excella E24 Incubator Shaker, New Brunswick Scientific) under air flow at 37 °C for 1 day. The samples were removed at fixed intervals (2 h) and weighed utilizing an electronic balance. The process was resumed until the samples demonstrated a constant weight (*w*1). The samples were then stored in distilled water and removed at intervals (0.5, 1, 2, 4, 8, 16, 24, and 36 h), blotted to remove surface water, weighed (*w*2), and again stored in water. This process was resumed until the samples demonstrated a constant weight. Three measurements were recorded for each formulation. The water sorption (%) was calculated according to the following equation:

Water Sorption = ((*w*2 − *w*1)/*w*1) × 100 (1)

### 4.7. Dynamic Mechanical Analysis (DMA) 

The rectangular beam samples were utilized to perform DMA temperature ramp experiments. Following the post-curing in the dark for 1 h at 23 ± 2 °C, the beam samples were submerged in distilled water at 37 °C for 2–4 days of aqueous aging. A Q800 DMA (TA Instruments, New Castle, DE, USA) mounted with a submersion 3-point bending clamp was employed [47]. The instrument was operated at a frequency of 1 Hz, displacement amplitude of 20 μm, and 0.001 N of pre-load force. The test was performed by ramping the temperature from ~10 °C to 75 °C at 1.5 °C min^−1^. The position of the maximum peak on the tan δ vs. temperature curve was utilized to determine the glass transition temperature (Tg). Three beam samples were tested for each formulation.

### 4.8. Characterizing Leached Species by High-Performance Liquid Chromatography (HPLC) 

Three transparent vials were each filled with 1 ml distilled water. Four rectangular beam samples post-cured in the dark for 1 h at 23 ± 2 °C were submerged in each vial at 37 °C to undergo 2, 3, and 4 days of aqueous aging. Extracts of 0.4 mL were removed at 2, 3, and 4 days to analyze HEMA concentrations by HPLC (Shimadzu LC-2010C HT, software Labsolutions 5.93). The HPLC system was incorporated with a packed column of 5 μm C-18 silica with a dimension of 250 × 4.6 mm (Luna, Phenomenex Inc., Torrance, CA, USA). A mixture of acetonitrile/water (60/40 *vol/vol*) was utilized as the mobile phase. The HPLC was operated under the following conditions: sample volume = 10 μL, flow rate = 0.5 mL min^−1^, detection at 208 nm, column temperature = 40 °C. The calibration of the system was performed by using standard HEMA solutions in water with concentrations of 2.5, 5, 10, 25, 50, 125, and 250 μg mL^−1^. We determined the HEMA concentration in the extracts by utilizing the calibration curve of standard HEMA solutions (Linear fitting of HEMA (2.5–125 μg mL^−1^, *R*^2^ = 0.9988)). The intensity of the HEMA peak at 6.3 min of retention time was utilized to calculate the concentrations. Note that three measurements were recorded for each extract.

### 4.9. Statistical Analysis

One-way analysis of variance (ANOVA, Microcal Origin Version 6.0, Microcal Software Inc., Northampton, MA, USA) was employed to determine the statistically significant differences in the mean values (*p* < 0.05).

## 5. Conclusions

In the present study, a self-strengthening hydrophilic adhesive with a relatively low crosslink density was designed. To mimic the load response in the wet oral environment, the mechanical properties were studied using water-saturated adhesive copolymers. The hydrophilicity of the adhesive combined with a relatively lower crosslink density could potentially maximize the free radical-polymerization and sol-gel reaction. Compared to a control adhesive without self-strengthening property, the aqueous-aged experimental adhesive demonstrated significantly improved mechanical properties as characterized by enhanced crosslink density. The HPLC studies also confirmed significantly lower HEMA leachate from the experimental adhesive, indicating an enhanced hydrolytic resistance. These self-strengthening formulations can offer promise as novel biomaterials with enhanced hydrolytic stability in the next generation of dental adhesives.

## Figures and Tables

**Table 1:** Values of degree of conversion (DC), maximum polymerization rate (*R_p_^max^*/[*M*]), water miscibility, and water sorption for control and experimental formulations.

Sample	DC (%) after 1 h	DC (%) after 24 h	*R_p_^max^*/[*M*] ×100 (s^−1^)	Water Miscibility (wt%)	Water Sorption (%)
Control	76.1 ^A^ (2.8)	84.3 ^A^ (5.8)	1.1 ^A^ (0.2)	25.8 ^A^ (0.4)	24.2 ^A^ (1.0)
Experimental	80.4 ^A^ (4.1)	88.3 ^A^ (1.4)	1.5 ^A^ (0.3)	24.6 ^B^ (0.2)	18.6 ^B^ (1.6)

The values in parentheses indicate the standard deviation. The same letter after the mean values indicates no statistically significant difference (*p* < 0.05).

**Table 2:** Dynamic mechanical analysis results of water-saturated copolymer samples in wet condition.

Sample	Storage Modulus at 37 °C (MPa)	Rubbery Modulus (MPa)	Tg (°C)	Tan δ Peak	ζ (×10^−5^ Pa^−1^ K)
C1-2 days	3.9 ^A^ (0.3)	2.7 ^A^ (0.1)	18.9 ^A^ (0.4)	1.02 ^A^ (0.01)	12.0 ^A^ (0.8)
C1-3 days	7.4 ^B^ (0.4)	4.3 ^B^ (0.1)	25.9 ^B^ (0.9)	0.93 ^B^ (0.01)	7.8 ^B^ (0.2)
C1-4 days	8.0 ^B^ (0.3)	4.5 ^B^ (0.3)	26.7 ^B^ (0.6)	0.94 ^B^ (0.01)	7.5 ^B^ (0.3)
E1-2 days	26.3 ^a^ (1.0)	9.0 ^a^ (0.2)	33.6 ^a^ (0.6)	0.72 ^a^ (0.01)	3.7 ^a^ (0.1)
E1-3 days	57.5 ^b^ (9.9)	12.8 ^b^ (1.0)	38.7 ^b^ (0.8)	0.65 ^b^ (0.01)	2.6 ^b^ (0.2)
E1-4 days	69.7 ^b^ (2.8)	13.3 ^b^ (0.4)	40.2 ^c^ (0.1)	0.63 ^b^ (0.00)	2.5 ^b^ (0.1)

The same letter after the mean values indicates no statistically significant difference (*p* < 0.05) within C1 (upper case letters) or E1 (lower case letters).

**Table 3:** Chemical composition of the formulations.

Components	C1 (wt%)	E1 (wt%)
HEMA	73	73
BisGMA	15	15
MES	10	-
MPS	-	10
CQ	0.5	0.5
EDMAB	0.5	0.5
DPIHP	1.0	1.0

## Data Availability

Not applicable.
